# The anterior cervical angle: A novel predictor of vaginal birth

**DOI:** 10.1002/ijgo.70396

**Published:** 2025-07-24

**Authors:** Erdal Şeker, Coşkun Ümit, Hasan Süt, Mustafa Koçar, Ecem Sarıtaş, Gülşah Aynaoğlu Yıldız

**Affiliations:** ^1^ Department of Obstetrics and Gynecology Ankara University School of Medicine Ankara Türkiye

**Keywords:** cesarean section, delivery, Transperineal ultrasound, utero‐cervical angle

## Abstract

**Objective:**

This study investigates the role of anterior utero‐cervical angle as a predictor of labor induction success, comparing outcomes between normal vaginal delivery and cesarean section groups.

**Methods:**

A prospective cohort study was conducted on 55 patients undergoing labor induction (46 normal vaginal delivery, 9 cesarean section). Parameters such as maternal age, weight, height, body mass index (BMI; calculated as weight in kilograms divided by the square of height in meters), cervical length, anterior cervical angle (ACA), Bishop score, and levator ani measurements were analyzed. Statistical significance was assessed using Fisher exact test, independent sample test, Mann–Whitney *U* test, or *χ* ^2^ test (*P* < 0.05).

**Results:**

The ACA was significantly different between normal vaginal delivery (108.02 ± 22.38°) and cesarean section (86.11 ± 26.09°) groups (*P* = 0.026). We determined an optimal ACA threshold of 96° for the prediction of failure of progression (sensitivity: 70%, specificity: 73%). Other significant differences included maternal weight (*P* = 0.0033), height (*P* = 0.0033), BMI (*P* = 0.0462), primiparity (*P* = 0.0161), and fetal gender (*P* = 0.0334). However, Bishop score, cervical length, and levator ani metrics showed no significant differences.

**Conclusion:**

Although ACA differs significantly between vaginal delivery and cesarean section groups, its independent predictive value for induction success remains limited compared with factors like primiparity and BMI. Further prospective studies are needed to clarify its clinical utility.

## 1 INTRODUCTION

Worldwide, the rate of cesarean section (CS) has risen from 7% to 21% and continues to increase. At the same time, the proportion of induced labor to all births has reached one‐fifth and is also on the rise.^1^ Factors contributing to the rising rates of CS include changes in professional training and practice, increasing fear of litigation, and changing social and cultural expectations, all of which are thought to lead to higher rates of CS during labor and at full dilation.^2^ Reasons for labor induction that contribute to the increased CS rates include maternal or fetal indications such as post‐term pregnancy, pre‐eclampsia, or fetal growth restriction.^3^ The success of labor induction, i.e. achieving a vaginal birth, depends on several factors, including cervical maturity (assessed by Bishop score), maternal characteristics, and fetal position.^4^ Despite advances in obstetric care, the rate of CS following failed inductions remains of great concern and prompts research into new predictors of induction outcome. Failed induction leading to CS is challenging for mother, baby, and medical staff, as both the induction process and the subsequent urgent surgery—often under emergency conditions—compound these difficulties. From this perspective, predicting induction failure and anticipating CS remain an important priority.

In the past, labor induction and its course were determined by the Bishop score.^5^, ^6^ However, the subjective nature of this scoring system has led to inconsistencies. Various parameters have been used to predict labor success, but these have led to inconsistent results across studies, with some relying on subjective assessments.^7^ To objectively assess the success and progress of labor, the use of ultrasound during labor has gained importance and has been included in guidelines.^8^, ^9^, ^10^, ^11^ Ultrasound parameters such as head to symphysis distance, angle of progression (AoP), and head to perineum distance measure the position of the fetal head, while studies on fetal head orientation and dynamic pelvic changes (e.g. levator ani and Valsalva‐induced AoP progression) have also been investigated.^12^

**FIGURE 1:**
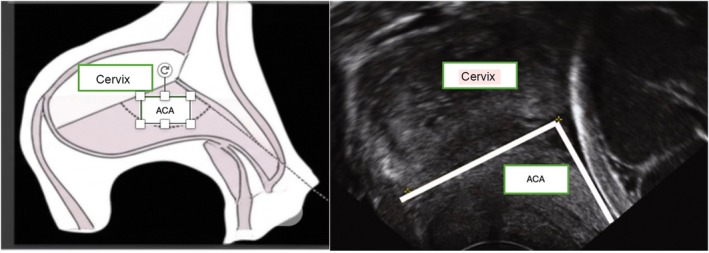
Schematic and ultrasonic representation of the anterior cervical angle.

In addition to the traditional ultrasound parameters, the anterior cervical angle (ACA) has become the focus of research. The ACA is the angle between a line along the cervix and another line along the lower anterior uterine segment extending from the internal os (Figure 1) It has been investigated for its role in predicting both successful induction of labor and preterm birth. In addition to conventional measurements, the ACA has also been associated with preterm birth.^13^ In addition, a few studies have looked at the use of ACA and posterior uterocervical angle measurements to predict labor induction success, although the literature is limited and conflicting. In this study we investigated the relationship between transperineal and transvaginal ultrasound measurements—before induction and during the early stages of labor—and vaginal delivery (VD) success, as they may contribute to increasing CS rates. We specifically examined the relationship between ACA and CS rates, including the likelihood of emergency CS during labor.

## 2 MATERIALS AND METHODS

**FIGURE 2:**
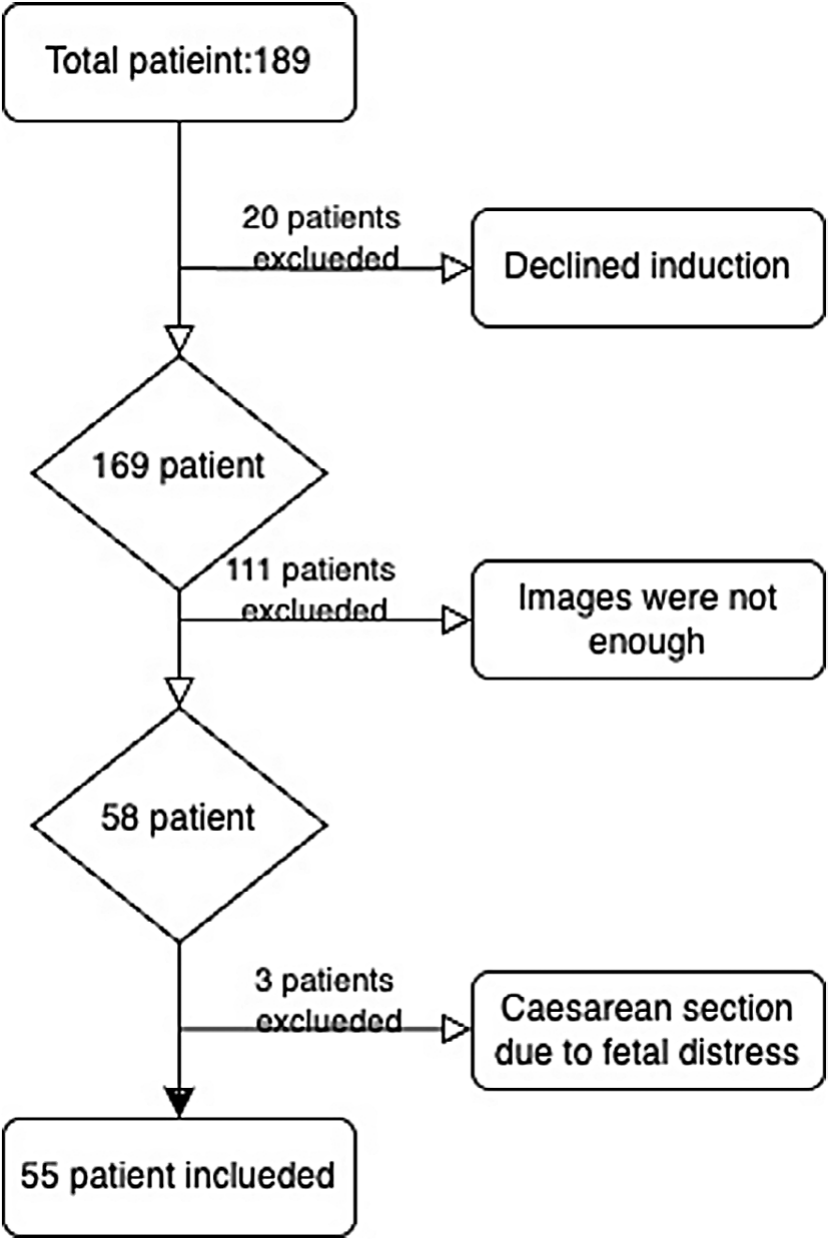
Flowchart of study.

This prospective cohort study included 189 pregnant women who underwent induction of labor or were in the latent phase of spontaneous labor at a single tertiary care center between January 2022 and March 2023. Our direct aim was to find a parameter that could predict VD. The latent phase refers to the period of slow cervical dilation, typically from 0 to 4 cm or 6 cm. We also included pregnant women in the latent phase, because most of those requiring induction and delivery are in this phase. Inclusion criteria were singleton pregnancies, cephalic location, and gestation between 36 and 42 weeks. Exclusion criteria included multiple pregnancies, breech presentation, or previous uterine surgery (e.g. myomectomy or previous CS). A total of 111 pregnant women were not included in the study because the images obtained by the doctors were insufficient, and the angles could not be measured. Three pregnant women were excluded from the study because of fetal distress during labor. This was because our objective was to identify cases of CS due to failed induction. After applying these criteria, 55 women were included in the study. The flow chart for inclusion and exclusion of participants is shown in Figure 2.

Maternal demographics (age, weight, height, body mass index [BMI; calculated as weight in kilograms divided by the square of height in meters]), obstetric history (primiparity), and pregnancy details (gestational age, estimated fetal weight, fetal sex) were obtained from medical records. All patients were informed on admission to hospital. Transvaginal, transabdominal, and transperineal ultrasound examinations were performed on the consenting participants. For fetal measurements, we performed transabdominal ultrasound. For parameters such as AoP and head to symphysis distance, we used transperineal ultrasound. We performed transvaginal ultrasound for cervical length and the ACA because these are best measured transvaginally in cases of head descent. All valid ultrasound scans were performed by second‐year perinatology subspecialty residents. All pregnant women in both latent and active labor phases were measured at their initial admission and during a period without contractions. Dynamic ultrasound measurements were performed three times at the time of peak Valsalva moment, with maximum and minimum values recorded. Ultrasound parameters included cervical length (CL), ACA, AoP at rest and during Valsalva, and levator ani muscle dimensions (at rest, Valsalva, and during contraction). The Bishop score was assessed by an experienced obstetrician at the beginning of labor induction. The ACA was measured by transvaginal ultrasonography as the angle between the cervical canal and the anterior uterine wall, following the methodology of Torkildsen et al..^14^ The aim of our study was to define and test for differences in ultrasound scan, examination, and demographic characteristics between pregnant women who had a VD and those who underwent CS. The demographic and ultrasonographic characteristics of the participants and fetuses are summarized in Table 1.

**TABLE 1:** Demographic and ultrasound data of pregnant women and fetuses.^a^

Characteristic	Vaginal delivery (*n* = 46; 81%)	Cesarean section (*n* = 9; 19%)	*P* value
Primiparity	15 (32%)	7 (77%)	0.024^b^
Multiparity	31 (67%)	2 (22%)	0.85
Age, years	27.98 ± 5.11 (18–44)	29.56 ± 4.64 (24–38)	0.395^c^
Weight. kg	75.02 ± 13.11 (58–108)	89 ± 12.35 (71–107)	0.003^d^
Height, cm	161.19 ± 5.13 (150–175)	166.55 ± 3.53 (160–170)	0.003^d^
Gestational week	39.39 ± 1.32 (36–42)	39.22 ± 1.09 (37–40)	0.690^d^
BMI	28.87 ± 4.12 (22.66–37.11)	32.02 ± 3.80 (26.93–38.37)	0.046^c^
Estimated fetal weight, g	3308.22 ± 504.12 (2275–4198)	3622.44 ± 332.97 (3196–4174)	0.072^d^
Gestational weight gain, kg	12.35 ± 6.28 (−10 to 30)	9.89 ± 6.91 (1–25)	0.329^c^
Cervical length, cm	23.57 ± 8.64 (6–44)	25.66 ± 8.89 (8–39)	0.439^d^
Cervical angle, %	108.02 ± 22.38 (40–152)	86.11 ± 26.09 (42–125)	0.026
Bishop score	4.93 ± 2.91 (0–11)	3.67 ± 3.04 (0–9)	0.241^c^
Occiput LOT	23 (50%)	5 (55.6%)	0.524^b^
AoP rest (angle), %	100.26 ± 15.05 (68–131)	99.67 ± 11.65 (82–119)	0.911^c^
AoP Valsalva (angle), %	112.91 ± 15.25 (78–138)	107.78 ± 10.30 (96–122)	0.339^c^
AoP differences, %	12.65 ± 11.05 (−20 to 30)	8.11 ± 6.84 (−2 to 18)	0.242^c^
Levator, rest, cm	68.96 ± 8.28 (48–83)	67.22 ± 9.49 (55–79)	0.577^c^
Levator, Valsalva, cm	71.22 ± 11.69 (43–93)	70.67 ± 12.14 (54–86)	0.898^c^
Levator, contraction, cm	57.37 ± 9.13 (41–80)	57.56 ± 9.87 (46–74)	0.956^c^
Levator differences (Valsalva–rest)	2.26 ± 10.43 (−22 to 20)	3.44 ± 6.12 (−8 to 13)	0.936^d^
Levator differences (rest–contraction)	11.58 ± 7.57 (−3 to 29)	9.66 ± 5.59 (1–22)	0.386^d^
Levator differences (Valsalva–contraction)	13.84 ± 11.89 (−7 to 40)	13.11 ± 9.26 (1–29)	0.862^c^
Birth weight, g	3253.52 ± 495.43 (2000–4345)	3482.22 ± 471.64 (2975–4380)	0.295^d^
Fetal sex (female)	23 (50%)	1 (11.1%)	0.033^e^
Occiput posterior	9 (16%)	0 (0%)	0.095
Latent phase, minute	11	4	0.236

Abbreviations: AoP, angle of progression; BMI, body mass index (calculated as weight in kilograms divided by the square of height in meters); LOT, left occiput transverse.

^a^ Data are presented as mean ± standard deviation (range), as number (percentage), or as number.

^b^ Fisher exact test.

^c^ Independent sample test.

^d^ Mann–Whitney *U* test.

^e^ Chi‐square test.

Continuous variables were expressed as means ± standard deviations and analyzed using the *t* test for independent samples (for normally distributed data) or the Mann–Whitney *U* test (for non‐normally distributed data). Categorical variables (e.g. primiparity, fetal sex) were assessed using Fisher exact test or *χ* ^2^ test. A value of *P* less than 0.05 was considered statistically significant. The relationship between cervical angle and birth outcome was assessed using a receiver operating characteristic curve. All statistical analyses were performed using SPSS version 26.0 (IBM Corp., Armonk, NY, USA).

The study was approved by the Institutional Review Board (IRB No. 2024‐03‐01) of the tertiary care center, and patient data were anonymized to ensure confidentiality. Informed consent was obtained from all participants.

## 3 RESULTS

Of the 55 pregnant women included, 9 (19%) underwent CS because labor did not progress, while 46 (81%) achieved VD, giving a CS rate of 16% for this indication. The mean age of the mothers was 27.98 ± 5.11 years in the group and 29.56 ± 4.64 years in the CS group (*P* = 0.3952), showing no significant difference. However, significant differences were observed in maternal weight (VD: 75.02 ± 13.11 kg, CS: 89 ± 12.35 kg; *P* = 0.0033), height (VD: 161.19 ± 5.13 cm, CS: 166.55 ± 3.53 cm; *P* = 0.0033) and BMI (VD: 28.87 ± 4.12, CS: 32.02 ± 3.80; *P* = 0.0462). Primiparity was significantly higher in the CS group (77.8%) than in the VD group (32.6%) (*P* = 0.0161). Gestational age (VD: 39.39 ± 1.32 weeks, CS: 39.22 ± 1.09 weeks; *P* = 0.6903) and estimated fetal weight (VD: 3308.22 ± 504.12 g, CS: 3622.44 ± 332.97 g; *P* = 0.0723) showed no significant differences.

**FIGURE 3:**
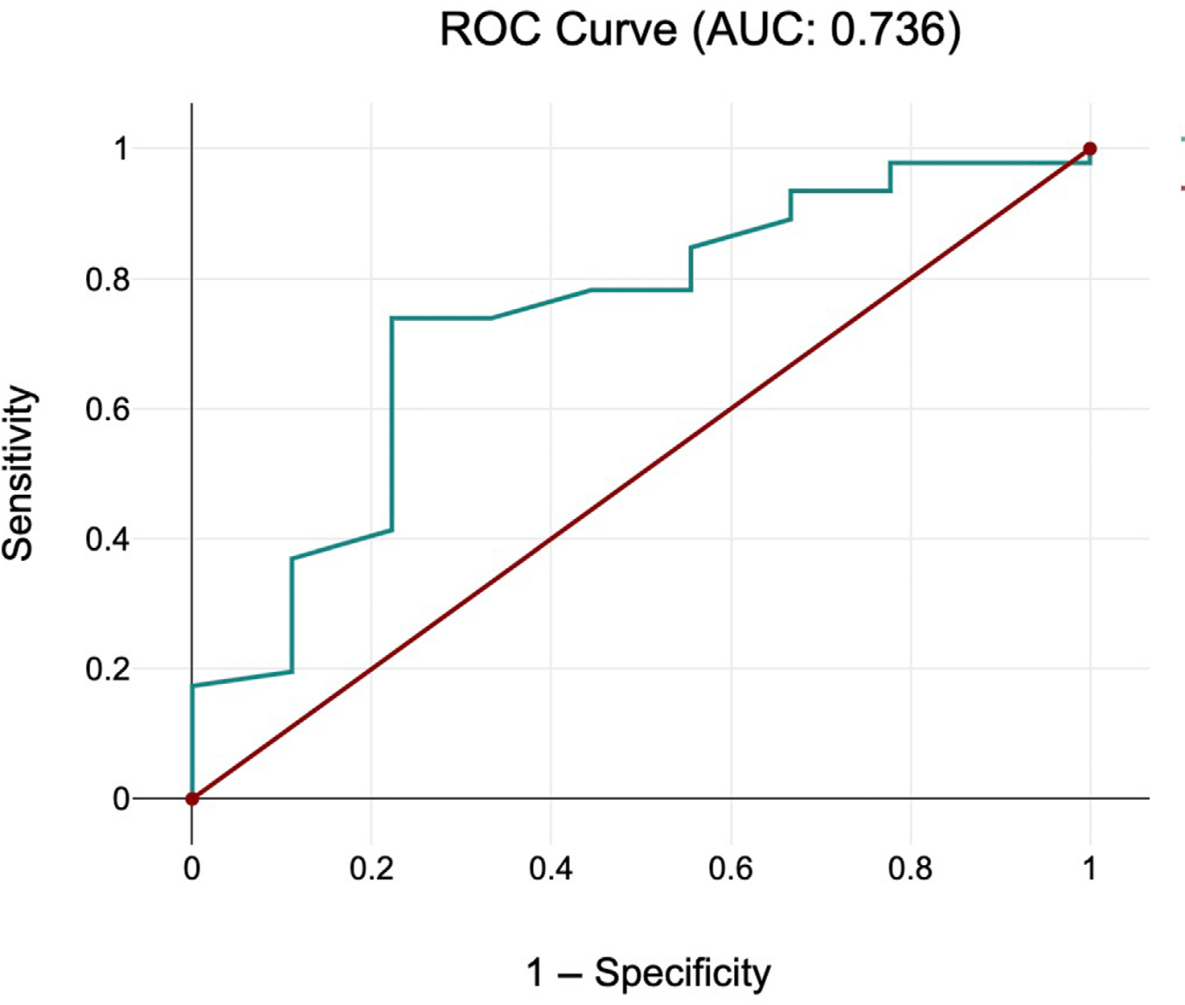
Receiver operating characteristics (ROC) area under the curve (AUC) analysis of anterior cervical angle.

In our study, CA was significantly lower in the CS group than in the VD group because of the absence of progression (86° versus 108°, *P* = 0.026). The optimal cut‐off point was determined using the Youden Index. We determined an optimal ACA threshold of 96° for the prediction of failure of progression (sensitivity: 70%, specificity: 73%). The receiver operating characteristics curve illustrating the relationship between the ACA and VD is shown in Figure 3. Cervical length (VD: 23.57 ± 8.64 mm, CS: 25.66 ± 8.89 mm; *P* = 0.4393) and Bishop score (VD: 4.93 ± 2.91, CS: 3.67 ± 3.04; *P* = 0.2412) did not differ significantly, suggesting that these traditional markers were less meaningful in this cohort.

The static and dynamic transperineal ultrasound measurements revealed the following: AoP at rest (VD: 100.26 ± 15.05°, CS: 99.67 ± 11.65°; *P* = 0.9112) and during Valsalva (VD: 112.91 ± 15.25°, CS: 107.78 ± 10.30°; *P* = 0.3392) showed no significant differences, nor did AoP (VD: 12.65 ± 11.05°, CS: 8.11 ± 6.84°; *P* = 0.2422). The measurements of the levator ani, including at rest (VD: 68.96 ± 8.28 mm, CS: 67.22 ± 9.49 mm; *P* = 0.5772), Valsalva (VD: 71.22 ± 11.69 mm, CS: 70.67 ± 12.14 mm; *P* = 0.8982) and contraction (VD: 57.37 ± 9.13 mm, CS: 57.56 ± 9.87 mm; *P* = 0.9562) were also comparable, indicating a minimal influence of pelvic floor dynamics on the results. Detailed demographic and ultrasonographic data can be found in Table 1.

The correlation analysis between ACA and delivery times among the 46 successful VD demonstrated a weak relationship with no statistical significance. These correlation data are presented in Table 2.

**TABLE 2:** Correlation analysis between cervical angle and stages of labor.

Variable	Pearson correlation (with anterior cervical angle)	Significance (two‐tailed)
Second stage of labor	−169	216
Active phase	0.322	16
Latent phase	32	817

## 4 DISCUSSION

Our study found a significant association between the ACA and the likelihood of VD, with larger angles correlating with a higher likelihood of successful VD. We have established an optimal ACA cut‐off of 96°, which is consistent with previous research.^15^ The ACA could act as a mechanical barrier in front of the cervix, modulating cervical strain and thereby influencing both preterm labor and vaginal birth. We initiated the study by incorporating numerous ultrasound scans, examinations, and demographic parameters to predict the success of VD. Among these parameters, only ACA was found to be significant. We believe that ACA changes with fetal descent and contributes to the biomechanics of labor. Several studies have shown the predictive value of ACA for labor outcome and preterm birth.^16^ In our cohort of VD, the mean ACA was 108.02 ± 22.38°, which is consistent with the angles reported in the literature in preterm labor.^17^

Compared with transvaginal ultrasound measurement of CL, ACA has been shown to have a higher sensitivity.^13^ When used together, their combined specificity reaches 99%. However, existing studies lack data on subsequent interventions (e.g. progesterone administration, cervical cerclage) that could influence the result, making it difficult to account for possible confounding factors. However, another study comparing the posterior cervical angle rather than CL or ACA found it to be a more significant predictor of vaginal birth outcomes.^15^

In a prospective study involving 159 women, transvaginal sonographic CL and ACA measurements taken between 18 and 24 weeks of pregnancy were assessed for their predictive value in preterm birth.^16^ ACA demonstrated greater sensitivity than CL, with a cut‐off value of 95°, which is consistent with our findings. However, that study reported a preterm birth rate of 28% (49 out of 159 deliveries before 37 weeks), which is notably higher than the rates reported by the World Health Organization.^18^ This discrepancy suggests that the study population may have consisted of a high‐risk cohort or may have been subject to selection bias.

Other statistically significant differences between groups included primiparity (*P* = 0.016), maternal weight (*P* = 0.003), height (*P* = 0.003), and fetal gender (*P* = 0.0334). We analyzed multiparous women separately and found no significant differences. The small sample size might have played a role in this. CS rates were higher among primiparous, shorter, and heavier women, with primiparity emerging as a strong predictor, consistent with previous literature. Nulliparous women, lacking previous experience of VD, are more likely to experience prolonged labor or failure to progress, thereby increasing the likelihood of CS.^19^, ^20^ The higher BMI observed in the CS group is in line with previous findings that identify obesity as a risk factor for labor dystocia and failed induction. Differences in maternal height and weight may reflect anthropometric influences on pelvic capacity, warranting further investigation. Additionally, female fetuses were associated with higher VD rates, a finding supported by literature suggesting that male fetuses are linked to increased labor difficulties and higher CS rates due to larger head circumference and birth weight.^21^, ^22^, ^23^ Insufficient placental steroidogenesis and reduced oxygenation capacity in male fetuses have also been proposed as contributing factors.^24^, ^25^

Studies examining levator muscle coactivation during Valsalva maneuvers have explored the relationship between levator length and the duration of the second stage of labor,^12^, ^26^ though findings remain inconclusive. While levator hiatus widening is typically expected with Valsalva, some studies have reported reductions associated with prolonged labor, potentially due to underlying pelvic pathologies such as endometriosis. Our study found no significant association between Valsalva‐induced levator changes and labor outcomes, possibly due to the higher baseline CS rates in Turkey and the broad indications for CS, which may obscure potential differences.^27^ In Turkey, the current medicolegal laws do not adequately protect doctors against financial and criminal proceedings. Consequently, doctors tend to avoid risky procedures. During labor, decisions for a CS are easily made in cases of prolonged labor, arrest of labor, or even at the family's request.

In comparison, the Bishop score did not demonstrate a statistically significant difference between groups (*P* = 0.2412), despite a slightly higher mean score in the NVD group (4.93 versus 3.67). This suggests that ACA may capture positional dynamics not accounted for by the Bishop score, which primarily evaluates cervical dilatation, effacement, and consistency.^5^ However, the predictive value of ACA appears to be weaker than that of maternal factors such as primiparity and BMI. The lack of statistical significance of the Bishop score in our study aligns with its known limitations as a subjective assessment tool.

This study has certain limitations, including the relatively small sample size and the inclusion of both primiparous and multiparous women. However, its strengths lie in its prospective, single‐center design and the exclusion of CS cases due to fetal distress, which enhances cohort homogeneity. Nonetheless, the single‐center approach may limit the generalizability of the findings, and unmeasured confounding variables, such as oxytocin dosage and indications for labor induction, may have influenced the results.

In conclusion, the present study demonstrates that the ACA differs significantly between the VD and CS groups following labor induction (*P* = 0.026), with wider angles being associated with an increased likelihood of VD. However, its predictive value is relatively limited when compared with stronger factors such as primiparity, BMI, and maternal anthropometric characteristics. Although ACA provides valuable insights into cervical positioning, its inherent variability and modest effect size constrain its standalone applicability in clinical practice. Future research should focus on prospective, multicenter studies with larger cohorts to further elucidate its clinical relevance and explore its integration with established predictive metrics. At present, ACA should be regarded as an adjunctive rather than a definitive predictor of induction success.

## AUTHOR CONTRIBUTIONS

EŞ and HS conceived and designed the study; EŞ, CÜ, MK, and ES collected and analyzed the data; and EŞ and GAY interpreted the results and drafted the manuscript. All authors approved the final version of the manuscript.

## CONFLICT OF INTEREST STATEMENT

The authors have no conflicts of interest.

## Data Availability

I will share the article if it is published.
